# Cellular sensitivity to UV-irradiation is mediated by RNA polymerase I transcription

**DOI:** 10.1371/journal.pone.0179843

**Published:** 2017-06-21

**Authors:** Robin Assfalg, Marius Costel Alupei, Maximilian Wagner, Sylvia Koch, Omar Garcia Gonzalez, Adrian Schelling, Karin Scharffetter-Kochanek, Sebastian Iben

**Affiliations:** Department of Dermatology and Allergic Diseases, University of Ulm, Ulm, Germany; Virginia Commonwealth University, UNITED STATES

## Abstract

The nucleolus has long been considered to be a pure ribosome factory. However, over the last two decades it became clear that the nucleolus is involved in numerous other functions besides ribosome biogenesis. Our experiments indicate that the activity of RNA polymerase I (Pol I) transcription monitors the integrity of the DNA and influences the response to nucleolar stress as well as the rate of survival. Cells with a repressed ribosomal DNA (rDNA) transcription activity showed an increased and prolonged p53 stabilisation after UVC-irradiation. Furthermore, p53 stabilisation after inhibition and especially after UVC-irradiation might be due to abrogation of the HDM2-p53 degradation pathway by ribosomal proteins (RPs). Apoptosis mediated by highly activated p53 is a typical hallmark of Cockayne syndrome cells and transcriptional abnormalities and the following activation of the RP-HDM2-p53 pathway would be a possible explanation.

## Introduction

Cockayne syndrome (CS) is a devastating autosomal recessive disease characterised by developmental and neurologic abnormalities, degeneration of several organ systems and sun sensitivity. CS is caused mainly by mutations in two genes: approximately 80% of all patients carry a mutation in the *CSB* gene (*ERCC6*) and 20% in the *CSA* gene (*ERCC8*). In addition, there is a subset of patients with mutations in the *XPB* (*ERCC3*), *XPD* (*ERCC2*) and *XPG* (*ERCC5*) gene that show somatic features of CS [1].

All genes have been identified to play an essential role in the DNA repair pathway nucleotide excision repair (NER). DNA repair by NER removes helix distorting lesions mainly caused by UV-light. CS is thought to be caused by a defect in the transcription coupled sub-pathway of NER (TC-NER) as the disease is mainly caused by mutations in the TC-NER specific *CSB* and *CSA* genes. Especially the sun sensitivity of CS patients seems to support this idea. However, it is difficult to explain the more severe symptoms with greater clinical significance (growth failure, developmental delay and neurological abnormalities) by a sole DNA repair dysfunction [2].

All five genes that cause CS have been shown to take part in Pol I transcription [3–7]. Thus, Pol I transcription and TC-NER are structurally linked. We hypothesise that there might be a functional link between Pol I transcription and UV-damage recognition. It has been shown that cells can overcome an immense amount of DNA damage without stabilising p53 as long as nucleoli are not disrupted [8]. Furthermore, not only DNA damage but also repression of Pol I transcription by knockdown of TIF-IA, as well as inhibition of ribosomal RNA (rRNA) synthesis by actinomycin D, induces nucleolar disruption followed by p53-dependent apoptosis [9–11].

To investigate whether a low Pol I transcription activity ensures a condition where additional transcriptional stress increases the probability of cell death, we simulated the specific rDNA transcription characteristics of CS cells in cells without any DNA repair defect. Our results show that Pol I transcription repression renders cells more prone to UVC-mediated apoptosis. Moreover, up-regulation of rRNA synthesis reduces the sensitivity to UVC-induced DNA damage. Our experiments clearly showed that the activity of Pol I transcription is monitored and influences the response to nucleolar stress as well as the rate of survival. Co-immunoprecipitation experiments unravelled that this p53 stabilisation is due to abrogation of the HDM2-p53 interaction. Interaction of ribosomal protein L11 with HDM2 after inhibition, UVC-irradiation or the combined treatment prevented p53 from degradation.

Apoptosis mediated by highly activated p53 is a typical hallmark of CS cells and of transcriptional abnormalities. Thus, the following activation of the RP-HDM2-p53 pathway would be a reasonable explanation.

## Material and methods

### Antibodies

**Table:** 

α p53	Abcam plc, Cambridge, United Kingdom / ab16465/immunoprecipitation
α p53	Abcam plc, Cambridge, United Kingdom / ab31333/Western blot
β-actin	Santa Cruz Biotechnology Inc., Heidelberg, Germany / sc-1615
α HDM2	Acris Antibodies GmbH, Herford, Germany / AM00224PU-N/Western blot
α HDM2	Santa Cruz Biotechnology Inc., Heidelberg, Germany / sc-7918/immunoprecipitation
rp L11	Proteintech group Inc. (16277-1-AP)

### Detection of apoptosis

Identification of a hypodiploid DNA content of young foreskin fibroblasts (FF95) and the Cockayne syndrome cell lines CS3BE and CS1AN after UVC-irradiation was performed as described by Nicoletti et al. in 1991 [12].

### Cell lines

CS1AN, CS3BE, FF95 and HCT116 cells were grown in Dulbecco’s Modified Eagle Medium with additional 10% foetal bovine serum, 2mM L-glutamine as well as 100U/ml penicillin and 100μg/ml streptomycin. CS1AN cells were a kind gift of Alan Lehmann, CS3BE of Mark Berneburg and HCT116 of Cagatay Guenes. FF95 primary fibroblasts were isolated and grown in the department of dermatology.

### qRT-PCR, primers, shRNA

Total RNA was isolated from exponentially growing cells using RNeasy kit (Qiagen) and reverse transcribed using random primer p(dN6) (Roche). Quantitative real time PCR was used to assess the expression levels of 47S (Forward primer 5’- TGTCAGGCGTTCTCGTCTC-3’, Reverse primer 5’- AGCACGACGTCACCACATC -3’), 5’ETS (Forward primer 5’- TGCGTGTCAGGCGTTCTCGTCTC-3’, Reverse primer 5’- TCACCACATCGATCGAAGAGCCC -3’), 5.8 ITS (Forward primer 5´-TCGTGCGTCGATGAAGAACGCAG-3´, Reverse primer 5´-ATTGATCGGCAAGCGACGCTCAG-3´), TIF-IA (Forward primer 5’- TGAGGCATGAAATTCTGGAGCTT-3’, Reverse primer 5’- CGTGGAATCTGTCCCACCAC -3’), RPL11 (Forward primer 5’- TGACCCAAGCATTGGTATCTACGG-3’, Reverse primer 5’- ATGGCCTCCTCTTTGCTGATTCTG -3’) and RPL13 (Forward primer 5’- CGGACCGTGCGAGGTAT-3’, Reverse primer 5’- CACCATCCGCTTTTTCTTGTC -3’). All primers were purchased from Thermo Fisher Scientific GmbH.

TIF-IA shRNA was purchased from Qiagen (336314KH01765P) SureSilencing shRNA Plasmid shRNA Clone ID: 5: CAACTTATCAGTATTATATTA; 6: CAATACTGGTGGAAAAATTTC; 7: CTTATTACTGTAAAATCATGC; 8: GGTCAAAGAAATTCATTGATC; NC: ggaatctcattcgatgcatac

### Northern blot

5μg of total RNA per lane was diluted in nuclease free water and mixed with the same volume of 2x RNA loading buffer. After denaturation for 15 minutes at 65°C, samples were chilled on ice for 5 minutes and separated by electrophoresis in 1x MOPS buffer on a 0.9% agarose gel for 45 minutes at 150V. The gel was stopped after 15 minutes and photographed to document the 28S- and 18S rRNA, which served as loading controls. After 45 minutes, the gel was blotted on a nylon membrane with 20x SSC buffer. Transfer was done overnight and RNA was cross-linked to the membrane on the next day with an UV Stratalinker™ 1800 (Stratagene, California, USA) using a dose of 0.24J. The membrane was blocked with 10ml of pre-warmed pre-hybridisation buffer for 2 hours at 68°C on a rotating wheel. The probe (template pGemHr) and was transcribed (T7 polymerase) for 1 hour at 37°C. 1μl of the RNA probe was diluted in 9μl probe loading buffer, loaded on a 4% TBE-polyacrylamide gel and run for 15 min at 320V. The gel was exposed to a phosphor-screen (Molecular Dynamics, GE Healthcare Europe GmbH, Freiburg, Germany) for 15 minutes, scanned with a Fujifilm FLA-300 (Fuji Photo Film Europe GmbH, Düsseldorf, Germany) and analysed using the BASReader 3.14 software and Aida Image Analyzer v.3.12 (Fuji Photo Film Europe GmbH, Düsseldorf, Germany). After an overnight hybridisation at 37°C, the membrane was washed twice with 10ml washing buffer for 10 minutes at 37°C and subsequently exposed to a phosphor-screen (Molecular Dynamics, GE Healthcare Europe GmbH, Freiburg, Germany) for 15 minutes.

### EU labeling of RNA

To assess the synthesis of new RNA transcripts after UV irradiation, cells were labeled with 5-ethynyl Uridine (EU) using Click-iT® Nascent RNA Capture Kit (Thermofischer Scientific) according to manufacturer’s instructions. Initially, cells were incubated with EU before beginning the treatment and harvested at the indicated time points. After incubation, total RNA or EU-labeled RNA was isolated as described in the previous section and used in a copper catalyzed click reaction with an azide-modified biotin, creating a biotin-based handle for capturing nascent RNA transcripts on streptavidin magnetic beads. The captured transcripts were used for reverse transcriptase-mediated cDNA synthesis and for subsequent analysis using qPCR.

### Inhibition of Pol I transcription

Pol I transcription was inhibited by either using actinomycin D, the specific inhibitor CX-5461 or transfection of TIF-IA specific short hairpin RNA.

TIF-IA specific shRNA: Plasmids containing TIF-IA specific shRNA were transfected by electroporation with a nucleofector device using an Amaxa™ Cell Line Nucleofector™ Kit according to the manufacturer’s instructions. Cells were selected for stable transfection by puromycin. To obtain a greater and more consistent level of knockdown, we performed single cell isolation of individual clones.

Actinomycin D: Pol I transcription was inhibited by a low concentration of actinomycin D. Therefore, media was supplemented with 10ng/ml of actinomycin D and cells were incubated for at least 6 hours before start of the experiments.

CX-5461: Cells were incubated with media containing 150nM of CX-5461 at least 2 hours before start of the experiments.

### Transfection

TIF-IA specific short hairpin RNA (shRNA) were transfected by electroporation with a nucleofector device using an Amaxa™ Cell Line Nucleofector™ kit according to the manufacturer’s instructions. Cells were selected for stable transfection with puromycin (1μg/ml). To obtain a greater and more consistent level of knockdown, individual clones from the pooled population were isolated. After increasing cell numbers, these monoclonal cell populations were screened for TIF-IA knockdown by qPCR as well as western blot analysis.

### Statistics

Data obtained from at least three independent experiments were analysed using the Graph Pad Prism 6 software. Data points were tested for normal distribution (D’Agostino-Pearson omnibus test) and for equality of variance (F test). Statistical differences of data from two sample groups were determined by using the unpaired t test (two tailed). Statistical differences of data from three or more groups were determined by repeated-measure analysis of variance (One-Way ANOVA) followed by Tukey or Dunnett’s post-test. Data of survival rates after UVC-irradiation were analysed by linear regression. Stars (*) in the figures represent ρ values (* = ρ<0.05, ** = ρ<0.01, *** = ρ<0.001).

## Approval

The study was approved by the ethical committee of ulm university (317/09).

## Results

### Stimulation of Pol I transcription initiation after UVC-irradiation

CS proteins are multifunctional proteins involved in repair of helix distorting DNA lesions as well as in Pol I transcription. Thus, these proteins provide a structural link between DNA repair and basal transcription, implying that DNA repair and basal transcription might also be functionally linked.

**Fig 1:**
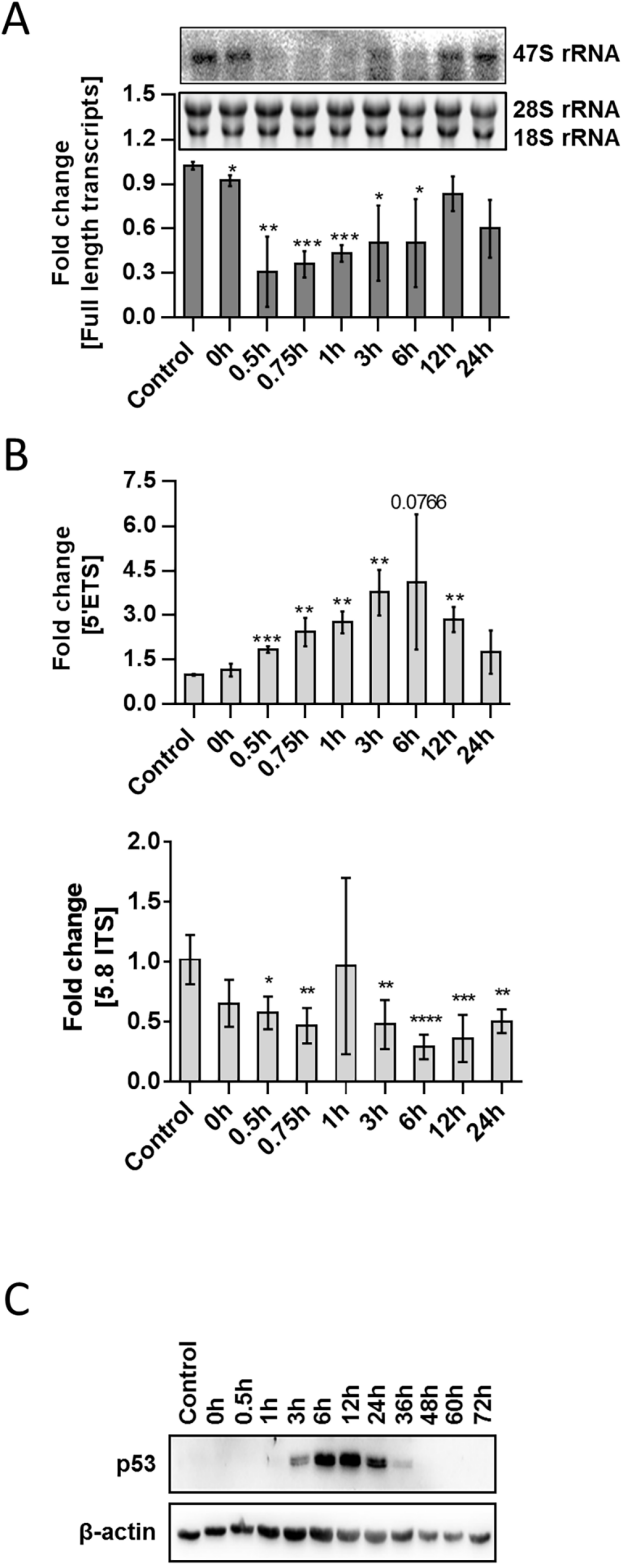
UVC-irradiation repressed the expression of full length transcripts (A) but stimulated Pol I transcription initiation (B). Young foreskin fibroblasts (FF95) with a cumulative population density under 25 were irradiated with 10J/m^2^ UVC and isolated RNA after the indicated time points was used for northern blot analysis (A) as well as qPCR amplifying the initiation (5`ETS) and elongation (5.8 ITS) sites of the pre-rRNA (B). (C) Whole cell lysates of irradiated (10J/m^2^) FF95 have been analysed for p53 stabilisation by western blot. Values are mean ± SD of three independent experiments (* = ρ<0.05, ** = ρ<0.01, *** = ρ<0.001). Blots are representatives of at least three independent experiments.

To investigate this hypothesis we asked the question how UV-light impacts Pol I transcription. Therefore, we performed UVC-irradiation kinetics of primary foreskin fibroblasts (FF95) and analysed transcription initiation and full length transcription of RNA polymerase I. As expected, full length transcription of the rDNA was repressed after irradiation which is shown by northern blot analysis (Fig 1A).

The repression was observed until 6 hours after irradiation and rDNA transcription recovers to normal level at 12–24 hours post irradiation. Mature rRNAs were not affected as revealed by ethidium-bromid staining of agarose gels indicating that ribosomal-bound rRNA might be stable throughout the timepoints of observation.

Surprisingly, the repression cycle seen for full length transcription was not reflected in transcription initiation by RNA polymerase I. QPCR analysis detecting the 5’ETS region of the rDNA revealed that there is an increased rDNA transcription initiation after UVC-irradiation starting already 0.5 hours post irradiation but a repression of elongation as detected by amplification of the 5.8S/ITS region (Fig 1B). To investigate if the production of all ribosome components is downregulated after UVC-irradiation, we analysed expression of ribosomal proteins. Therefore, we examined the expression of ribosomal protein L11 (RPL11) and ribosomal protein L13a (RPL13a) from kinetics of UVC-irradiated FF95 cells by qPCR and Western blot. As shown in S1B Fig, expression of these two ribosomal proteins did not decrease upon UVC-irradiation. As p53 levels are also mediated by nucleolar stress, we analysed p53 stabilisation after UVC-irradiation. Whole cell lysates of UVC-irradiation kinetics revealed stabilisation of p53 starting at 3 hours, with a maximum peak at 12 hours and a recovery to control levels 36–48 hours post irradiation (Fig 1C). UVC-irradiation leads to an increase in Pol I transcription initiation with concomitant increase in abortive rRNA transcripts (S1D Fig) as well as stabilization of p53. With a time lag the levels of p53 decrease when full-length transcription by RNA polymerase I recovers…

### Pol I transcriptional activity mediates UV-sensitivity

**Fig 2:**
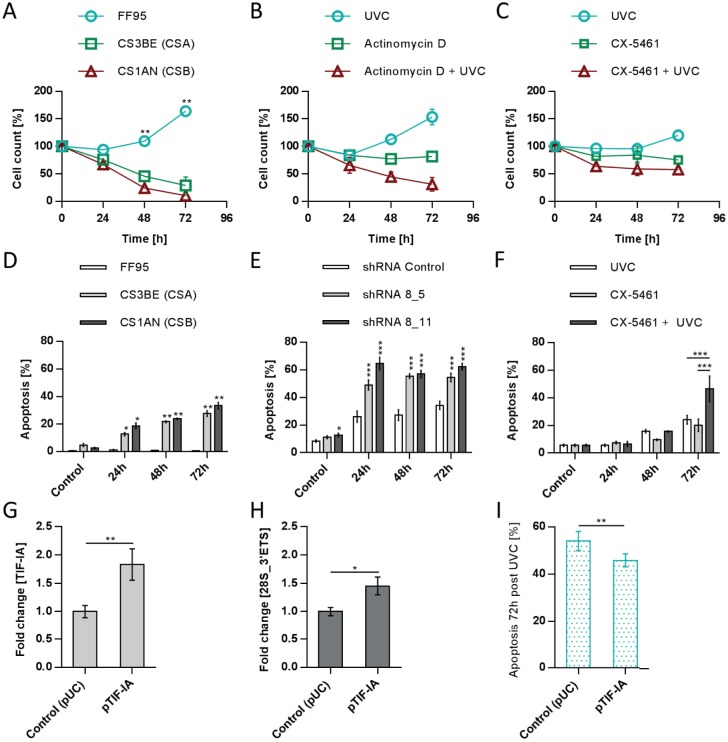
Repression of Pol I transcription by inhibitors rendered cells more sensitive to UVC-mediated DNA damage. Survival rate of cells with different Pol I transcription activity after UVC-irradiation. FF95 and CS cells were UVC-irradiated (10J/m^2^) and cell survival was measured by counting (A). Primary fibroblasts were treated with low-dose (10ng/ml) actinomycin D (B) or HCT116 cells with 150nM CX-5461 (C) in combination with UVC-irradiation (10J/m^2^). (D) Cells with a repressed Pol I transcription undergo apoptosis more frequently after UVC-irradiation. Identification of a hypodiploid DNA content of FF95, CS3BE (CSA) and CS1AN (CSB) after UVC-irradiation (Nicoletti). CS cells showed a significant increase in apoptosis after UVC-irradiation (10J/m^2^) (D). Inhibition of rDNA transcription in HCT116 cells by knockdown of TIF-IA (E) as well as by the specific compound CX-5461 (F) increased apoptotic cell death significantly. (G-I) HCT116 cells transfected with TIF-IA (G) show a significant increase in rRNA synthesis (H) and less apoptosis (I) after UVC-irradiation. (10J/m^2^). Values are mean ± SD of three independent experiments (* = ρ<0.05, ** = ρ<0.01, *** = ρ<0.001).

To examine whether cells with an impaired rDNA transcription react differently to UVC-mediated DNA damage, we irradiated CS as well as healthy cells and analysed cell survival rates. Healthy FF95 began to grow afresh after a delay of 24h, whereas CSA-mutated CS3BE cells and CSB-mutated CS1AN cells of CS patients did not survive (Fig 2A).

As CS cells show disturbances in Pol I transcription resulting in a decreased production of precursor rRNA, we mimicked this aspect by repressing Pol I transcription in healthy cells without any DNA repair defect. Low dose of actinomycin D, preferentially targeting Pol I transcription [13] led to growth inhibition in FF95 primary fibroblasts whereas additional treatment with UVC did not only lead to growth stagnation but death (Fig 2B). We also repressed rDNA transcription with the specific inhibitor CX-5461. CX-5461 works in cells with accelerated rDNA transcription and ribosomal biogenesis [14,15], thus we used the human colon carcinoma cell line 116 (HCT 116). Repression of Pol I transcription by CX-5461 (S1E and S1F Fig)) raised the sensitivity of HCT 116 cells towards UVC-irradiation as depicted in Fig 2C. To investigate whether Pol I-inhibited cells undergo apoptosis after UVC-irradiation, we used a variant of the propidium iodide flow cytometric assay (Nicoletti assay). First, we performed the nicoletti assay with UVC-irradiated FF95, CS3BE (CSA/CS) and CS1AN (CSB/CS) cells. As shown in Fig 2D, CS cells have a significantly increased apoptotic rate upon UVC-irradiation (10J/m^2^). In the next experiments, we measured the apoptotic rate of non-CS cells (HCT116) with a repressed rDNA transcription after UVC-irradiation (10J/m^2^). Although the transfection of control shRNA sensitised cells towards apoptosis induced by UVC-irradiation, specific repression of Pol I transcription by knockdown of TIF-IA as well as by the inhibitor CX-5461 led to a significant increased apoptosis rate after UVC-irradiation, demonstrating that the UV-sensitivity of cells directly correlates with the transcriptional activity of Pol I (Fig 2E and 2F). These results were further corroborated by experiments showing that activation of Pol I transcription by overexpression of TIF-IA (Fig 2G and 2H) decreases the UV-sensitivity of these cells as shown by a decreased apoptosis rate after UVC-irradiation (Fig 2I).

### P53 stabilisation after UVC-irradiation is mediated by ribosomal proteins

**Fig 3:**
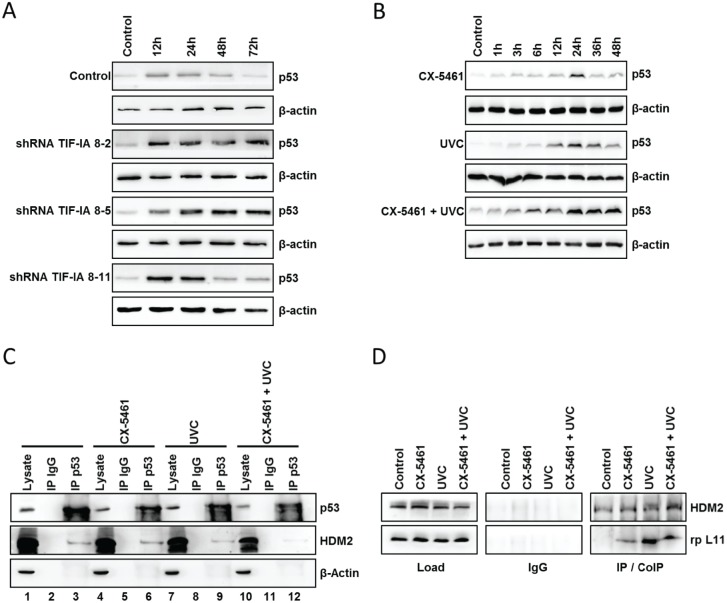
(A) TIF-IA silencing by shRNA led to increased and prolonged p53 levels after UVC-irradiation. Stable transfected HCT116 cells have been irradiated with 10J/m^2^ UVC and analysed for p53 stabilisation by western blot. (B) P53 levels in whole cell lysates of HCT116 cells treated with the specific Pol I inhibitor CX-5461 and irradiated with 10J/m^2^ UVC. (C, D) CoIP experiments of HCT116 cells treated with either CX-5461 or UVC-irradiation or a combination of both. Cells were first incubated with 5μM of MG132 for 12h. Afterwards one group was treated with 150nM CX-5461, a second group was irradiated with 10J/m^2^ UVC and the third group was treated with 150nM CX-5461 as well as irradiated with 10J/m^2^ UVC. Whole cell lysates were prepared 24h after UVC-irradiation and subsequently used for CoIP. Precipitated and co-precipitated proteins were analysed on western blots. (C) IP of p53 and CoIP of HDM2. (D) IP of HDM2 and CoIP of ribosomal protein L11 (rp L11). Blots are representatives of at least three independent experiments.

The integrity of nucleoli, regulated by ribosomal biogenesis, controls the steady state of p53 [8–11]. To investigate whether repression of rDNA transcription by knockdown of TIF-IA or the inhibitor CX-5461 is followed by p53 stabilisation, whole cell lysates of the respective treated HCT116 cells after UVC-irradiation have been prepared and analysed by western blot. Control cells showed a slight increase in p53 stabilisation between 12h to 24h after irradiation (Fig 3A).

P53 stabilisation after UVC-irradiation in cells with a repressed Pol I transcription was significantly increased and prolonged as shown by knockdown of TIF-IA (Fig 3A) and administration of CX-5461 (Fig 3B). Disturbances in Pol I transcription are followed by an excess of free ribosomal proteins that might inhibit HDM2 and therefore, stabilise p53 [16–21]. If the observed p53 increase after Pol I transcription inhibition is mediated by an increase of free ribosomal proteins was tested by performing CoIP experiments. To facilitate precipitation of possible p53 complexes, we enriched p53 levels in HCT116 cells by using the proteasome inhibitor MG132 (5μM) which reduces the degradation of ubiquitin-conjugated proteins. After 12h we performed single treatment of CX-5461 and UVC-irradiation, as well as a combination of both. Whole cell lysates were prepared 24h after UVC-irradiation and subsequently used for CoIP experiments. Western blot analysis of p53 precipitation samples using a HDM2 antibody showed that HDM2 was co-precipitated with p53 (Fig 3C, row 3). UVC-irradiation clearly resulted in a reduction of HDM2-p53 complexes (Fig 3C, row 9) whereas treatment with CX-5461 prior to UVC-irradiation resulted in an almost complete abrogation of the p53-HDM2 interaction (Fig 3C, row 12). To investigate whether this abrogation of HDM2-p53 interaction is at least in part due to inhibition of HDM2 by ribosomal proteins, we performed additional CoIP experiments. As shown in Fig 3D, western blot analysis revealed that RPL11 can be co-precipitated with HDM2 after all treatments. Interaction of RPL11 with HDM2 could already be observed in samples treated with CX-5461. UVC-irradiation increased the amount of RPL11 associated with HDM2. RPL11 could also be detected associated with HDM2 after combination of both stresses indicating that p53 stabilisation is at least partially mediated by competition of HDM2 binding by RPL11.

## Discussion

Blockage of RNA polymerase II transcription by DNA damage leads to a transcription stress response which may play an important role in safeguarding the genome [22]. However, there is no evidence that this mechanism should be restricted to RNA polymerase II transcription. In fact, protein coding genes make up only about 1–2% of the genome. A further candidate for a “DNA scanning device” is Pol I transcription. Ribosomal genes are distributed on five chromosomes, the human genome contains about 400 copies, transcription of these genes accounts for up to 60% of total transcription and the size of a single gene is 13kb. Thus the probability of a DNA damage event and its detection by Pol I is high. Induction of severe DNA damage is accompanied by a global repression of rDNA transcription in trans as revealed by a recent seminal report [23]. If ribosomal transcription is also repressed by UV-damage is less well characterized yet.

In this work, we first showed that UVC-irradiation of healthy foreskin fibroblasts (FF95) increases the initiation rate of Pol I transcription up to 4 fold, whereas expression of full length transcripts was severely inhibited. Pol I might stall at UVC-induced DNA lesions and thereby full length transcription is impaired. Alternatively, Pol I might fall off the rDNA at such blocking lesions and could initiate again at the rDNA promoter. This is in line with the observations made in yeast, that UV-irradiation is followed by an enhanced promoter occupancy of Pol I and the transcription initiation factor CF [24], thus allowing increased initiation. This increased initiation produces rRNA transcripts of increasing length over the time course of DNA repair (S1D Fig). As a result, the rDNA is scanned until lesions are repaired.

CS cells are characterised by an elevated UV-sensitivity and a reduced Pol I transcription [3–7]. Asking if these characteristics are causally linked, we performed irradiation experiments with CS cells and cells with a repressed Pol I transcription. Repression of rDNA transcription by different approaches rendered cells more sensitive to UV-induced cell death, a typical hallmark of CS cells. Moreover, up-regulation of rDNA transcription by transfection of TIF-IA significantly reduced the rate of apoptosis after UVC-irradiation. Taken together, the activity of Pol I transcription directly influences cell survival.

Looking for the central cell cycle and apoptosis regulator p53, that regulates cellular fate after nucleolar stress [8], we observed that a decrease in Pol I transcription by different approaches leads to an increased and prolonged stabilisation of p53 after UVC-irradiation. The increased and prolonged p53 stabilisation seems to be dependent on the fact that the repressed rRNA synthesis alters the stress tolerance of these cells. Stabilisation of p53 upon interaction of RPs with HDM2 was addressed by CoIP experiments. Treatment of CX-5461 as well as UVC-irradiation went along with the abrogation of HDM2-p53 interaction. This was due to increased binding of RPL11 with HDM2 and as a consequence, p53 was stabilised. The fact that RPL11 co-precipitated with HDM2 after UVC-irradiation indicates that UV-mediated stress is sensed by the balance of the ribosomal components. The decrease of full length rRNA transcripts leads to an excess of non-bound ribosomal proteins that are then stabilising p53.

The inability of cells with a repressed Pol I transcription activity to handle additional stress is supporting the idea that the nucleolus is a stress sensor safeguarding the integrity of our genome. Furthermore, in accordance to Rubbi et al. [8], we could show that inhibition of rRNA synthesis already led to stabilisation of p53 via the RP-HDM2-p53 pathway, even in the absence of DNA damage. In this regard, it seems that cells sense transcription abnormalities rather than DNA lesions themselves.

Apoptosis mediated by highly activated p53 is a typical hallmark of CS cells [25]. Whereas TC-NER provides a good explanation for the sun sensitivity of CS patients, it fails to explain the neurodevelopmental disorders as well as growth abnormalities and the progeroid features [2]. An alternative explanation for many CS characteristics would be transcriptional abnormalities and the following activation of the RP-HDM2-p53 pathway leading to elevated levels of p53. Cockayne syndrome cells are known to have elevated p53 levels and an autopsy of a 23-year-old woman with Cockayne syndrome revealed the correlation between Cockayne syndrome pathology and the expression patterns of p53 [26]. However, a reduced threshold for apoptosis does not explain the whole pathophysiology of Cockayne syndrome. There needs to be a second trigger to push these cells into apoptosis and this might not be UV-light as organs and tissues degenerate in this syndrome that are not affected by light.

## Supporting information

S1 Fig (A) EU-labelled RNA after UV irradiation shows increase in expression of 47S, as shown by qPCR analysis. (B) RNA of UVC-kinetics used in Fig 1A was further analysed by qPCR. Ribosomal protein L11 (rp L11) and ribosomal protein L13a (rp L13a) are not differently expressed after UVC-irradiation. Protein levels assessed by western blot also show no change after UVC-irradiation. (D) Northern blot analysis of UVC-irradiated skin fibroblasts. Smear at 0.5h to 12h post irradiation shows abortive rRNA transcripts until full length transcription recovers. Repression of Pol I transcription initiation was analysed by qPCR (C). (E) Repression by CX-5461 inhibited Pol I transcription in a dose-dependent manner and was stable over the time period of 50h (F). (H) Repression of Pol I transcription by transfection of TIF-IA specific shRNA. Western blot and qPCR analysis show the expression level of TIF-IA on RNA and protein level after stable transfection. (I) Quantification of IP of p53 and CoIP of HDM2 from Fig 3C. (J)Quantification of IP of HDM2 and CoIP of ribosomal protein L11 (rp L11) from Fig 3D. Values are mean ± SD of three independent experiments (* = ρ<0.05, ** = ρ<0.01, *** = ρ<0.001). Blots are representatives of at least three independent experiments.
(ZIP)
