# Supplementary material for: Cellular sensitivity to UV-irradiation is mediated by RNA polymerase I transcription
Source: PLoS One. 2017 Jun 21;12(6):e0179843. doi: 10.1371/journal.pone.0179843 (PMC5479586; doi:10.1371/journal.pone.0179843)
Supplement: S1 Fig — (A) EU-labelled RNA after UV irradiation shows increase in expression of 47S, as shown by qPCR analysis. (B) RNA of UVC-kinetics used in Fig 1A was further analysed by qPCR. Ribosomal protein L11 (rp L11) and ribosomal protein L13a (rp L13a) are not differently expressed after UVC-irradiation. Protein levels assessed by western blot also show no change after UVC-irradiation. (D) Northern blot analysis of UVC-irradiated skin fibroblasts. Smear at 0.5h to 12h post irradiation shows abortive rRNA transcripts until full length transcription recovers. Repression of Pol I transcription initiation was analysed by qPCR (C). (E) Repression by CX-5461 inhibited Pol I transcription in a dose-dependent manner and was stable over the time period of 50h (F). (H) Repression of Pol I transcription by transfection of TIF-IA specific shRNA. Western blot and qPCR analysis show the expression level of TIF-IA on RNA and protein level after stable transfection. (I) Quantification of IP of p53 and CoIP of HDM2 from Fig 3C. (J)Quantification of IP of HDM2 and CoIP of ribosomal protein L11 (rp L11) from Fig 3D. Values are mean ± SD of three independent experiments (* = ρ<0.05, ** = ρ<0.01, *** = ρ<0.001). Blots are representatives of at least three independent experiments. (ZIP) [file pone.0179843.s001.zip › FiguresPlosOne Supplemental fig.pptx]

## Slide 1
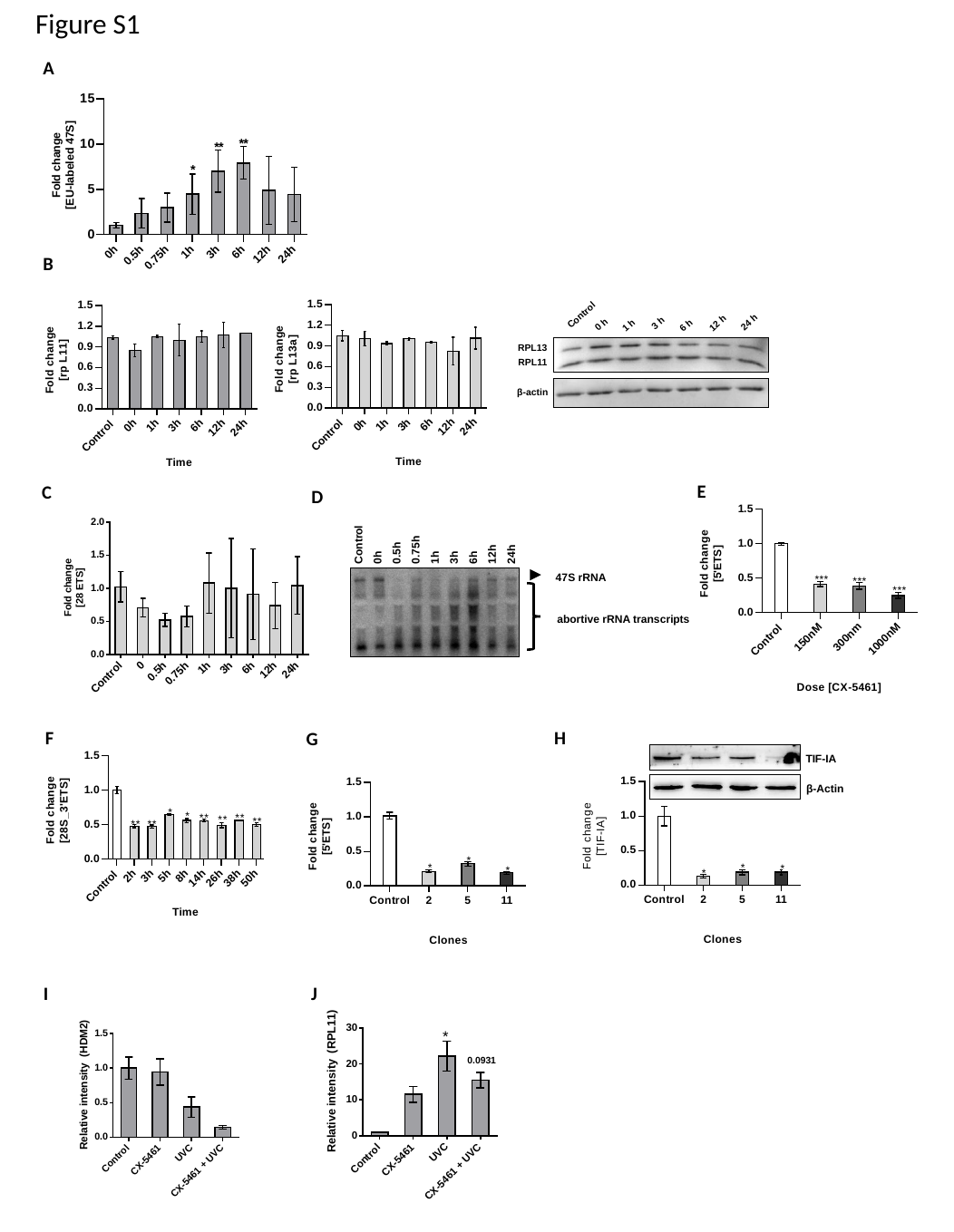

Figure S1
A
B
Control
24 h
3 h
12 h
0 h
6 h
1 h
RPL13
RPL11
β-actin
E
C
D
Control
0.75h
0.5h
12h
24h
0h
1h
3h
6h
47S rRNA
abortive rRNA transcripts
F
H
G
TIF-IA
β-Actin
I
J
*
0.0931

## Slide 2
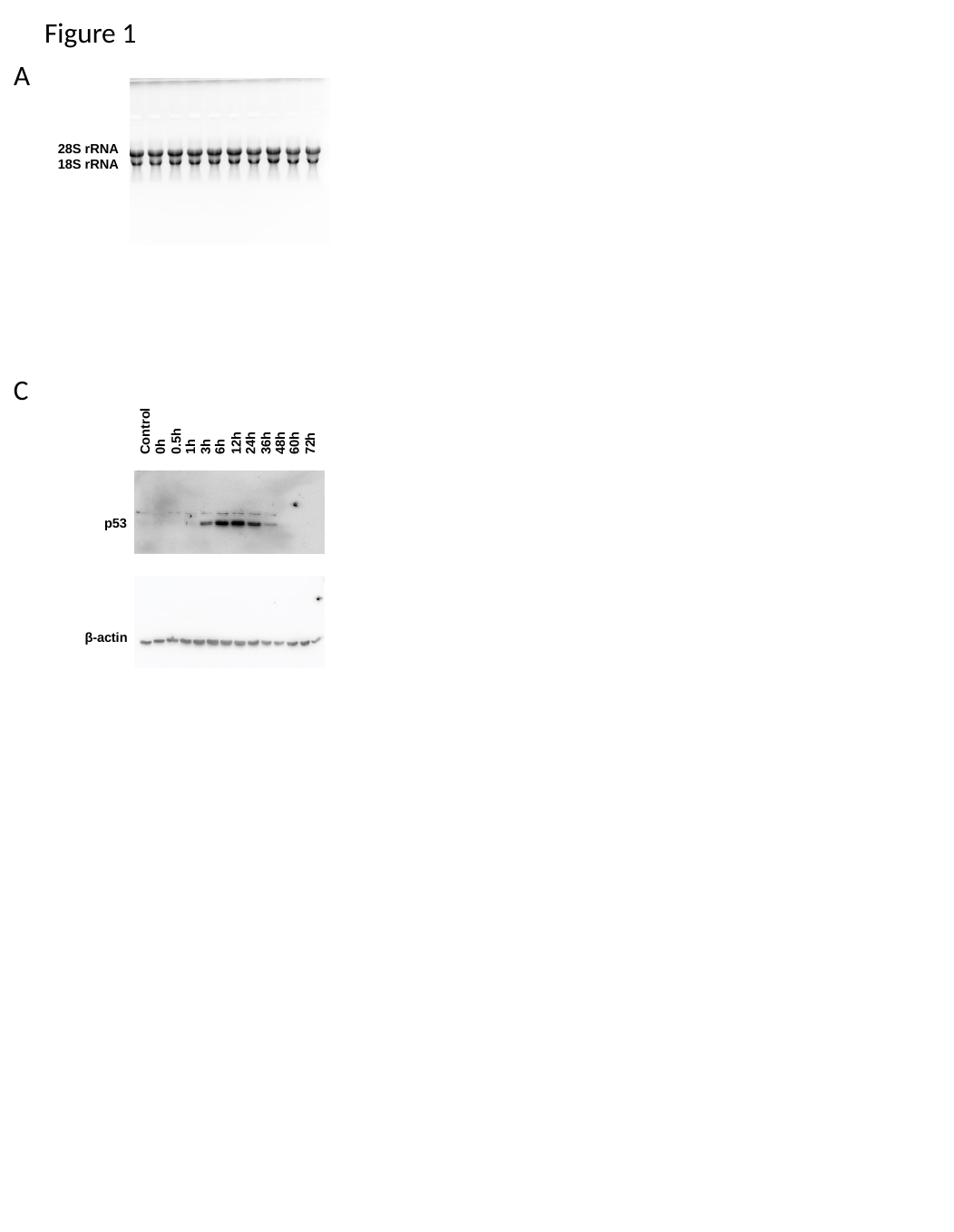

Figure 1
A
28S rRNA
18S rRNA
C
Control
0.5h
12h
24h
36h
48h
60h
72h
0h
1h
3h
6h
p53
β-actin

## Slide 3
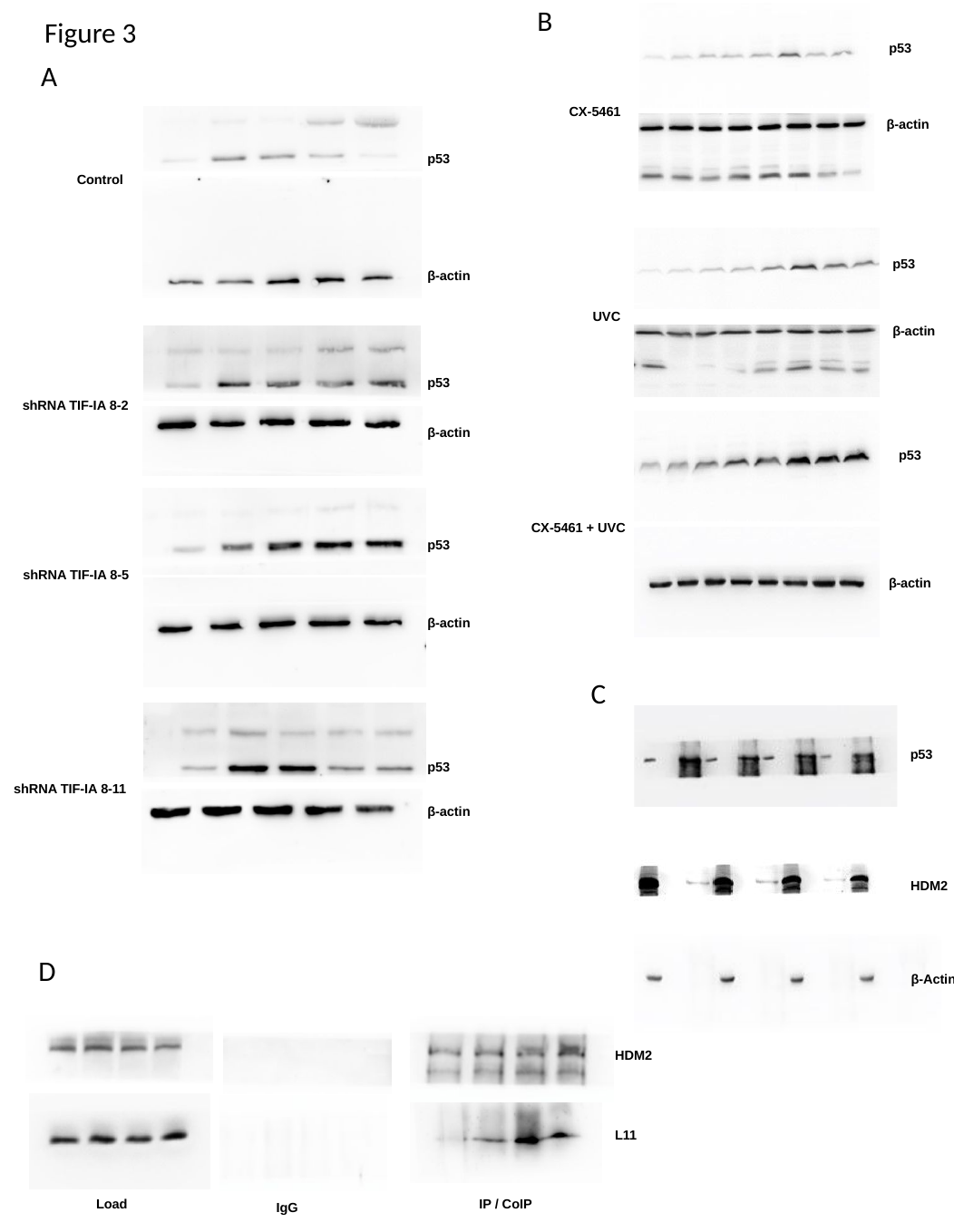

B
Figure 3
p53
A
CX-5461
β-actin
p53
Control
p53
β-actin
UVC
β-actin
p53
shRNA TIF-IA 8-2
β-actin
p53
CX-5461 + UVC
p53
shRNA TIF-IA 8-5
β-actin
β-actin
C
p53
p53
shRNA TIF-IA 8-11
β-actin
HDM2
D
β-Actin
HDM2
L11
Load
IP / CoIP
IgG

## Slide 4
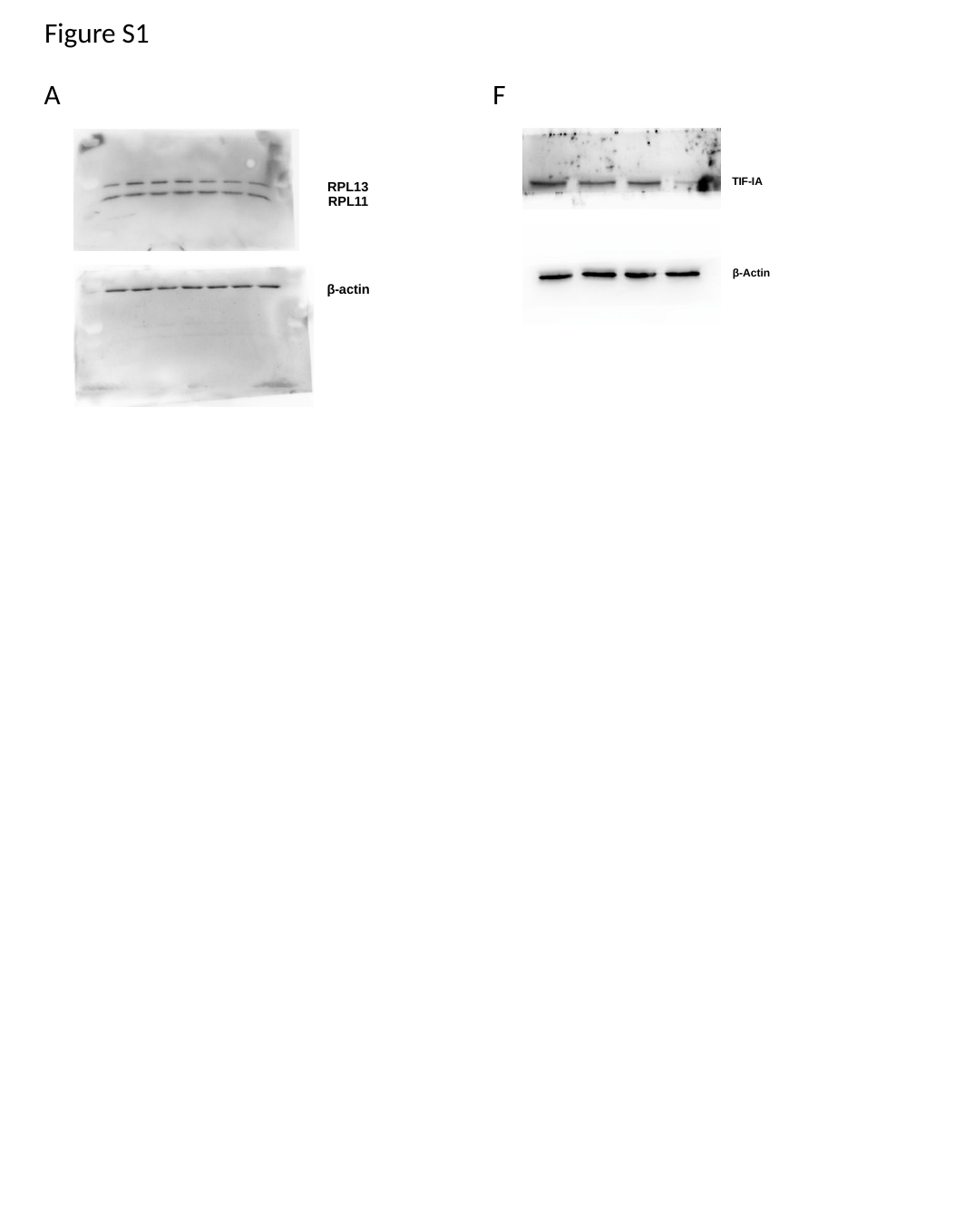

Figure S1
A
F
TIF-IA
RPL13
RPL11
β-Actin
β-actin

## Slide 5
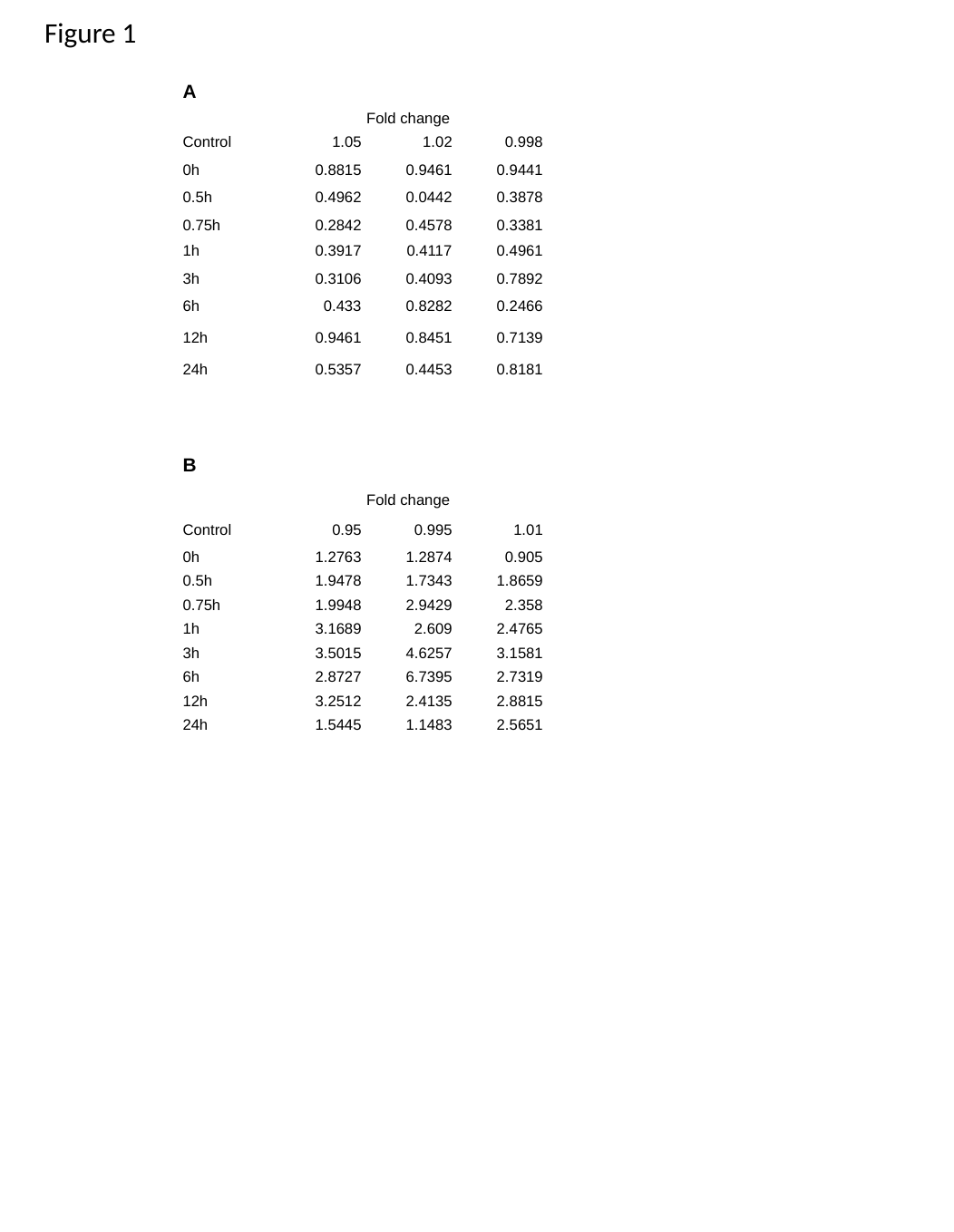

Figure 1
| | A | | | |
| --- | --- | --- | --- | --- |
| | | Fold change | | |
| | Control | 1.05 | 1.02 | 0.998 |
| | 0h | 0.8815 | 0.9461 | 0.9441 |
| | 0.5h | 0.4962 | 0.0442 | 0.3878 |
| | 0.75h | 0.2842 | 0.4578 | 0.3381 |
| | 1h | 0.3917 | 0.4117 | 0.4961 |
| | 3h | 0.3106 | 0.4093 | 0.7892 |
| | 6h | 0.433 | 0.8282 | 0.2466 |
| | 12h | 0.9461 | 0.8451 | 0.7139 |
| | 24h | 0.5357 | 0.4453 | 0.8181 |
| | | | | |
| | | | | |
| | B | | | |
| | | Fold change | | |
| | Control | 0.95 | 0.995 | 1.01 |
| | 0h | 1.2763 | 1.2874 | 0.905 |
| | 0.5h | 1.9478 | 1.7343 | 1.8659 |
| | 0.75h | 1.9948 | 2.9429 | 2.358 |
| | 1h | 3.1689 | 2.609 | 2.4765 |
| | 3h | 3.5015 | 4.6257 | 3.1581 |
| | 6h | 2.8727 | 6.7395 | 2.7319 |
| | 12h | 3.2512 | 2.4135 | 2.8815 |
| | 24h | 1.5445 | 1.1483 | 2.5651 |

## Slide 6
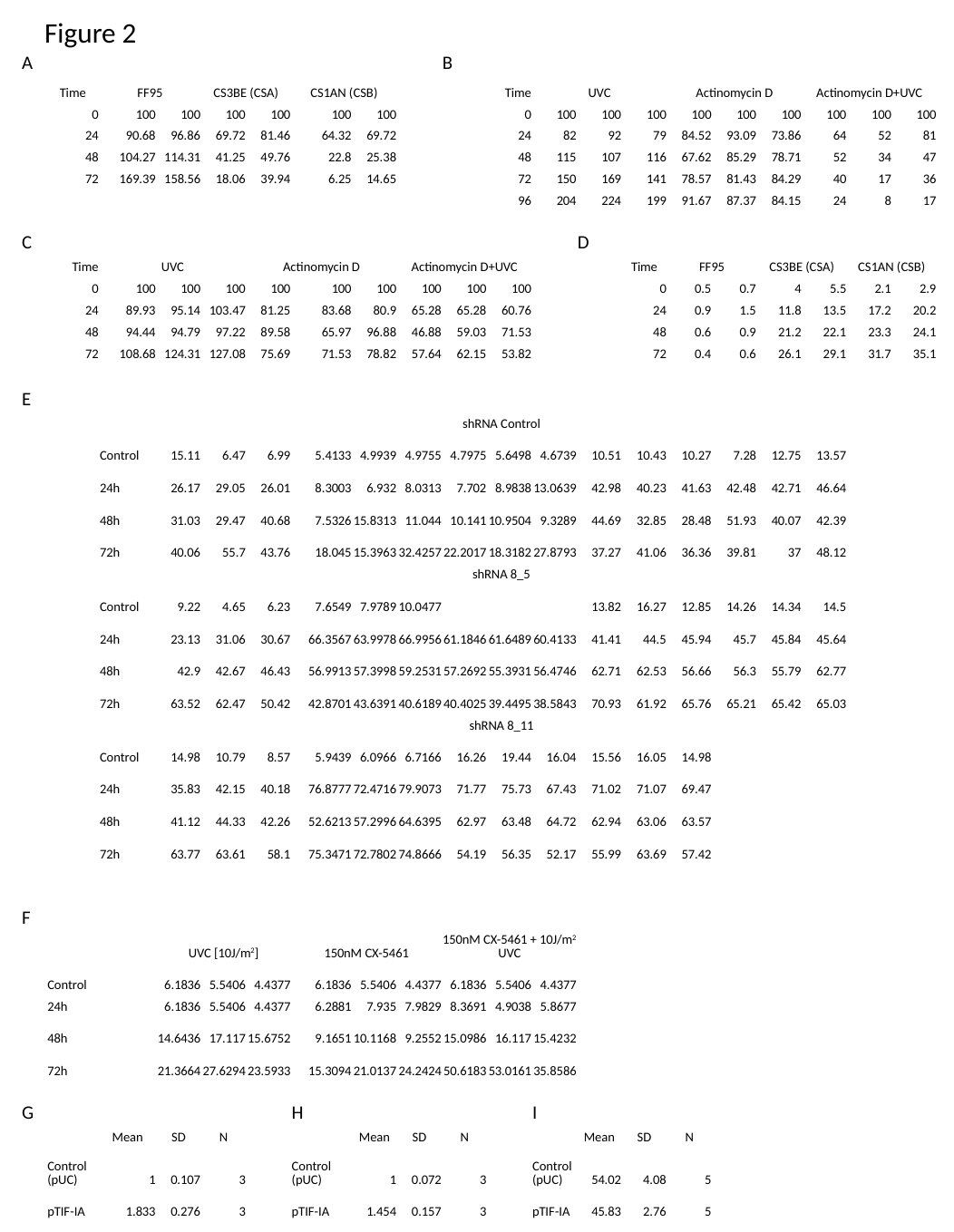

Figure 2
| A | | | | | | | | | B | | | | | | | | | | |
| --- | --- | --- | --- | --- | --- | --- | --- | --- | --- | --- | --- | --- | --- | --- | --- | --- | --- | --- | --- |
| | Time | FF95 | | CS3BE (CSA) | | CS1AN (CSB) | | | | Time | UVC | | | Actinomycin D | | | Actinomycin D+UVC | | |
| | 0 | 100 | 100 | 100 | 100 | 100 | 100 | | | 0 | 100 | 100 | 100 | 100 | 100 | 100 | 100 | 100 | 100 |
| | 24 | 90.68 | 96.86 | 69.72 | 81.46 | 64.32 | 69.72 | | | 24 | 82 | 92 | 79 | 84.52 | 93.09 | 73.86 | 64 | 52 | 81 |
| | 48 | 104.27 | 114.31 | 41.25 | 49.76 | 22.8 | 25.38 | | | 48 | 115 | 107 | 116 | 67.62 | 85.29 | 78.71 | 52 | 34 | 47 |
| | 72 | 169.39 | 158.56 | 18.06 | 39.94 | 6.25 | 14.65 | | | 72 | 150 | 169 | 141 | 78.57 | 81.43 | 84.29 | 40 | 17 | 36 |
| | | | | | | | | | | 96 | 204 | 224 | 199 | 91.67 | 87.37 | 84.15 | 24 | 8 | 17 |
| | | | | | | | | | | | | | | | | | | | |
| C | | | | | | | | | | | | D | | | | | | | |
| | Time | UVC | | | Actinomycin D | | | Actinomycin D+UVC | | | | | Time | FF95 | | CS3BE (CSA) | | CS1AN (CSB) | |
| | 0 | 100 | 100 | 100 | 100 | 100 | 100 | 100 | 100 | 100 | | | 0 | 0.5 | 0.7 | 4 | 5.5 | 2.1 | 2.9 |
| | 24 | 89.93 | 95.14 | 103.47 | 81.25 | 83.68 | 80.9 | 65.28 | 65.28 | 60.76 | | | 24 | 0.9 | 1.5 | 11.8 | 13.5 | 17.2 | 20.2 |
| | 48 | 94.44 | 94.79 | 97.22 | 89.58 | 65.97 | 96.88 | 46.88 | 59.03 | 71.53 | | | 48 | 0.6 | 0.9 | 21.2 | 22.1 | 23.3 | 24.1 |
| | 72 | 108.68 | 124.31 | 127.08 | 75.69 | 71.53 | 78.82 | 57.64 | 62.15 | 53.82 | | | 72 | 0.4 | 0.6 | 26.1 | 29.1 | 31.7 | 35.1 |
| | | | | | | | | | | | | | | | | | | | |
| E | | | | | | | | | | | | | | | | | | | |
| | | | shRNA Control | | | | | | | | | | | | | | | | |
| | | Control | 15.11 | 6.47 | 6.99 | 5.4133 | 4.9939 | 4.9755 | 4.7975 | 5.6498 | 4.6739 | 10.51 | 10.43 | 10.27 | 7.28 | 12.75 | 13.57 | | |
| | | 24h | 26.17 | 29.05 | 26.01 | 8.3003 | 6.932 | 8.0313 | 7.702 | 8.9838 | 13.0639 | 42.98 | 40.23 | 41.63 | 42.48 | 42.71 | 46.64 | | |
| | | 48h | 31.03 | 29.47 | 40.68 | 7.5326 | 15.8313 | 11.044 | 10.141 | 10.9504 | 9.3289 | 44.69 | 32.85 | 28.48 | 51.93 | 40.07 | 42.39 | | |
| | | 72h | 40.06 | 55.7 | 43.76 | 18.045 | 15.3963 | 32.4257 | 22.2017 | 18.3182 | 27.8793 | 37.27 | 41.06 | 36.36 | 39.81 | 37 | 48.12 | | |
| | | | shRNA 8\_5 | | | | | | | | | | | | | | | | |
| | | Control | 9.22 | 4.65 | 6.23 | 7.6549 | 7.9789 | 10.0477 | | | | 13.82 | 16.27 | 12.85 | 14.26 | 14.34 | 14.5 | | |
| | | 24h | 23.13 | 31.06 | 30.67 | 66.3567 | 63.9978 | 66.9956 | 61.1846 | 61.6489 | 60.4133 | 41.41 | 44.5 | 45.94 | 45.7 | 45.84 | 45.64 | | |
| | | 48h | 42.9 | 42.67 | 46.43 | 56.9913 | 57.3998 | 59.2531 | 57.2692 | 55.3931 | 56.4746 | 62.71 | 62.53 | 56.66 | 56.3 | 55.79 | 62.77 | | |
| | | 72h | 63.52 | 62.47 | 50.42 | 42.8701 | 43.6391 | 40.6189 | 40.4025 | 39.4495 | 38.5843 | 70.93 | 61.92 | 65.76 | 65.21 | 65.42 | 65.03 | | |
| | | | shRNA 8\_11 | | | | | | | | | | | | | | | | |
| | | Control | 14.98 | 10.79 | 8.57 | 5.9439 | 6.0966 | 6.7166 | 16.26 | 19.44 | 16.04 | 15.56 | 16.05 | 14.98 | | | | | |
| | | 24h | 35.83 | 42.15 | 40.18 | 76.8777 | 72.4716 | 79.9073 | 71.77 | 75.73 | 67.43 | 71.02 | 71.07 | 69.47 | | | | | |
| | | 48h | 41.12 | 44.33 | 42.26 | 52.6213 | 57.2996 | 64.6395 | 62.97 | 63.48 | 64.72 | 62.94 | 63.06 | 63.57 | | | | | |
| | | 72h | 63.77 | 63.61 | 58.1 | 75.3471 | 72.7802 | 74.8666 | 54.19 | 56.35 | 52.17 | 55.99 | 63.69 | 57.42 | | | | | |
| | | | | | | | | | | | | | | | | | | | |
| | | | | | | | | | | | | | | | | | | | |
| F | | | | | | | | | | | | | | | | | | | |
| | | | UVC [10J/m2] | | | 150nM CX-5461 | | | 150nM CX-5461 + 10J/m2 UVC | | | | | | | | | | |
| | Control | | 6.1836 | 5.5406 | 4.4377 | 6.1836 | 5.5406 | 4.4377 | 6.1836 | 5.5406 | 4.4377 | | | | | | | | |
| | 24h | | 6.1836 | 5.5406 | 4.4377 | 6.2881 | 7.935 | 7.9829 | 8.3691 | 4.9038 | 5.8677 | | | | | | | | |
| | 48h | | 14.6436 | 17.117 | 15.6752 | 9.1651 | 10.1168 | 9.2552 | 15.0986 | 16.117 | 15.4232 | | | | | | | | |
| | 72h | | 21.3664 | 27.6294 | 23.5933 | 15.3094 | 21.0137 | 24.2424 | 50.6183 | 53.0161 | 35.8586 | | | | | | | | |
| | | | | | | | | | | | | | | | | | | | |
| G | | | | | | H | | | | | I | | | | | | | | |
| | | Mean | SD | N | | | Mean | SD | N | | | Mean | SD | N | | | | | |
| | Control (pUC) | 1 | 0.107 | 3 | | Control (pUC) | 1 | 0.072 | 3 | | Control (pUC) | 54.02 | 4.08 | 5 | | | | | |
| | pTIF-IA | 1.833 | 0.276 | 3 | | pTIF-IA | 1.454 | 0.157 | 3 | | pTIF-IA | 45.83 | 2.76 | 5 | | | | | |

## Slide 7
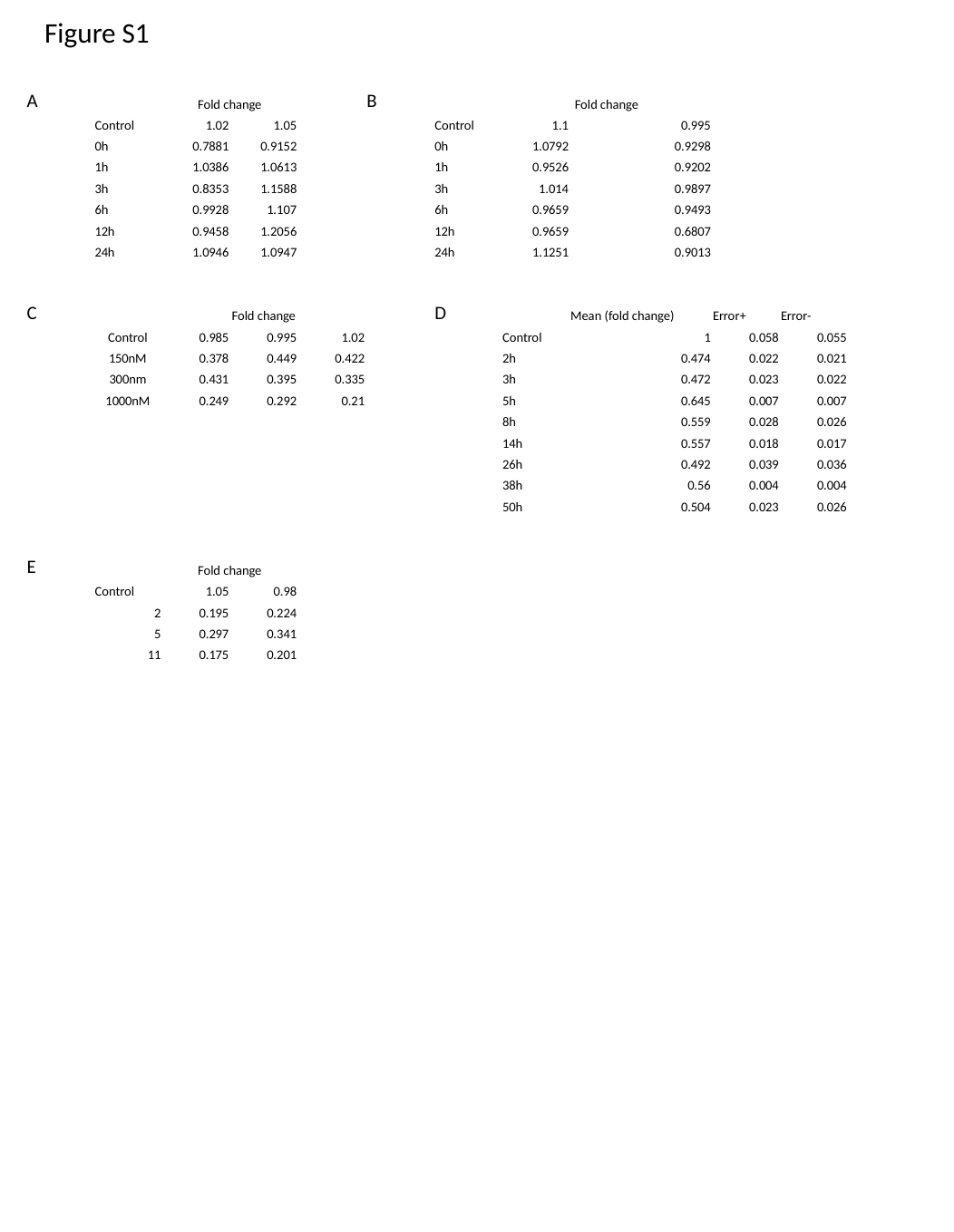

Figure S1
| A | | Fold change | | | B | | Fold change | | | |
| --- | --- | --- | --- | --- | --- | --- | --- | --- | --- | --- |
| | Control | 1.02 | 1.05 | | | Control | 1.1 | 0.995 | | |
| | 0h | 0.7881 | 0.9152 | | | 0h | 1.0792 | 0.9298 | | |
| | 1h | 1.0386 | 1.0613 | | | 1h | 0.9526 | 0.9202 | | |
| | 3h | 0.8353 | 1.1588 | | | 3h | 1.014 | 0.9897 | | |
| | 6h | 0.9928 | 1.107 | | | 6h | 0.9659 | 0.9493 | | |
| | 12h | 0.9458 | 1.2056 | | | 12h | 0.9659 | 0.6807 | | |
| | 24h | 1.0946 | 1.0947 | | | 24h | 1.1251 | 0.9013 | | |
| | | | | | | | | | | |
| | | | | | | | | | | |
| C | | Fold change | | | | D | | Mean (fold change) | Error+ | Error- |
| | Control | 0.985 | 0.995 | 1.02 | | | Control | 1 | 0.058 | 0.055 |
| | 150nM | 0.378 | 0.449 | 0.422 | | | 2h | 0.474 | 0.022 | 0.021 |
| | 300nm | 0.431 | 0.395 | 0.335 | | | 3h | 0.472 | 0.023 | 0.022 |
| | 1000nM | 0.249 | 0.292 | 0.21 | | | 5h | 0.645 | 0.007 | 0.007 |
| | | | | | | | 8h | 0.559 | 0.028 | 0.026 |
| | | | | | | | 14h | 0.557 | 0.018 | 0.017 |
| | | | | | | | 26h | 0.492 | 0.039 | 0.036 |
| | | | | | | | 38h | 0.56 | 0.004 | 0.004 |
| | | | | | | | 50h | 0.504 | 0.023 | 0.026 |
| | | | | | | | | | | |
| | | | | | | | | | | |
| E | | Fold change | | | | | | | | |
| | Control | 1.05 | 0.98 | | | | | | | |
| | 2 | 0.195 | 0.224 | | | | | | | |
| | 5 | 0.297 | 0.341 | | | | | | | |
| | 11 | 0.175 | 0.201 | | | | | | | |
